# Caregiving Across Diverse Populations: New Evidence From the National Study of Caregiving and Hispanic EPESE

**DOI:** 10.1093/geroni/igz033

**Published:** 2019-09-09

**Authors:** Sunshine M Rote, Jacqueline L Angel, Heehyul Moon, Kyriakos Markides

**Affiliations:** 1 Kent School of Social Work, University of Louisville, Kentucky; 2 School of Public Affairs and Department of Sociology, The University of Texas at Austin, Galveston; 3 Preventive Medicine & Community Health, University of Texas Medical Branch, Galveston

**Keywords:** Caregiving, Ethnicity, Informal, Minority issues, Well-being

## Abstract

**Background and Objectives:**

The current study employs population-based data to determine the extent to which stress and coping factors are related to self-rated health and distress for informal caregivers (CGs) from the 3 largest racial/ethnic groups in the United States (non-Latino White, African American, and Mexican American).

**Research Design and Methods:**

Data on primary, informal CGs are obtained from the 2015 National Study of Caregiving (NSOC) (*n* = 667) and the 2016 Hispanic Established Populations for the Epidemiologic Studies of the Elderly (H-EPESE) CG supplement (*n* = 287). Logistic regression models of health are presented for all CGs and specifically for dementia CGs.

**Results:**

Caregiving intensity is related to health for non-Latino White CGs and African American dementia CGs. Support from family and friends is related to better self-rated health, but only for African American dementia CGs. While better relationship quality is related to better health for African American CGs and White dementia CGs, formal support utilization is related to worse CG health for Mexican American dementia CGs.

**Discussion and Implications:**

Findings emphasize the importance of earlier detection and intervention with CGs at the beginning in the caregiving career, the interplay of formal and informal support, and appropriate ways to intervene with dementia CGs. Culturally tailored home- and community-based care options are needed to supplement the low levels of CG support, especially for the Mexican American population.

**Translational Significance**

Interventions for racial/ethnic minority caregivers at the microlevel should focus on fostering social support, appreciation, and reciprocity earlier in the caregiving career, while policies should enable increased access to and availability of culturally responsive home- and community-based formal support options.

## Background and Objectives

More than 17 million people in the United States are informal, unpaid caregivers (CGs) to an older adult (National Academies of Sciences, Engineering, and Medicine, 2016). Informal CGs help their care recipient/s (CR/CRs) with many tasks, including household, transportation, medical and self-care support (National Academies of Sciences, Engineering, and Medicine, 2016). On average, CGs spend approximately 24 hr per week helping with such tasks, and their economic contribution to the U.S. economy estimated to be close to $470 billion, with dementia CGs contributing over $41 billion of that sum (Family Caregiver Alliance, 2016; Rabarison et al., 2018). Informal care, especially informal dementia care, is more prevalent and more intense for racial/ethnic minority families than non-Latino White families (Mehta & Yeo, 2019; Rote & Moon, 2018). Currently, about 30% of family CGs self-identify as a racial/ethnic minority, and this percentage is expected to increase in the upcoming years as the older adult population becomes more diverse (National Academies of Sciences, Engineering, and Medicine, 2016).

Population-based studies with representation from the largest racial/ethnic groups in the United States are limited; this is mostly due to lack of statistical power for multiple racial/ethnic groups in data on CGs (National Academies of Sciences, Engineering, and Medicine, 2016). Generally, population-based studies tend to report more positive aspects of caregiving, including benefits to health and well-being (e.g., Haley et al., 2009; Roth, Fredman, & Haley, 2015b). Our study contributes to this body of research by examining factors related to informal, primary CG health for CGs from the three largest racial/ethnic groups in the United States (African American, non-Latino White, and Mexican American), regardless of CG–CR relationship type (child, spouse, etc.) or CR health condition (stroke, dementia, etc.). Given possible racial/ethnic differences in expressions of stress, we include psychological health and self-rated health as key dependent variables. We also include important background and confounding factors in our study that determine individual health status (Roth et al., 2015b; Schulz & Sherwood, 2008).

We draw on two unique data sets, the National Study of Caregiving (NSOC) and the Hispanic Established Populations for the Epidemiologic Studies of the Elderly (H-EPESE) CG supplement. Using the CG stress and coping process to guide our study, we examine the extent to which caregiving intensity, CG support (formal and informal support), CG–CR relationship quality, and CG background factors (relationship type, age, education, income, gender) are related to informal CG health (self-rated health and distress) for non-Latino White, African American, and Mexican American CGs. Given the substantial and unique challenges in dementia care, we also investigate whether the same factors are related to health for non-Latino White, African American, and Mexican American dementia CGs.

### Caregiving Stress and Coping Processes

Like other complex social roles, caregiving involves both rewards and costs. The vast majority of CGs generally report a positive experience, so long as the responsibilities of caring for others do not become excessive relative to the resources available (Beach, Schulz, Yee, & Jackson, 2000). The population over the age of 85 is increasing, and CGs—who are aging themselves—are likely to suffer from their own serious health problems, decreasing the ability to cope with the increased CG burden (Taylor & Quesnel-Vallée, 2017). Previous research has established major sources of CG stress and their mental health consequences (Horwitz & Reinhard, 1995). CGs who perceive fewer benefits and greater role strain report more depression and lower life satisfaction (Haley, LaMonde, Han, Burton, & Schonwetter, 2003). There are also differences in CG health and well-being, stressors, and support by race/ethnicity.

The stress process model, which draws from theories of stress and coping, is the most commonly used framework in studies on CG health (Pearlin, Mullan, Semple, & Skaff, 1990; Zarit, Femia, & Whitlatch, 2016). It has been extended to the sociocultural stress and coping model which focuses specifically on racial/ethnic differences in CG health (Aranda & Knight, 1997; Knight & Sayegh, 2010). In this model, race/ethnicity shapes CG health, and influences caregiving intensity, support, appraisals, and background factors.

### Race/Ethnicity and CG Health

Existing studies demonstrate that African American and Latino CGs report lower levels of depression and higher levels of life and CG satisfaction, on average, in comparison to non-Latino White CGs (Dilworth-Anderson, Williams, & Gibson, 2002; Knight & Sayegh, 2010; Roth, Dilworth-Anderson, Huang, Gross, & Gitlin, 2015a). This finding has been attributed to cultural values that place emphasis on the family unit and family-based support. However, there is also evidence that racial/ethnic minority CGs report worse self-rated health and negative physical health over time (Pinquart & Sorensen, 2005; Roth, Haley, Owen, Clay, & Goode, 2001), suggesting that caregiving stressors may manifest in poor physical health for racial/ethnic minority CGs. Caregiving stressors within in the CG stress process model include caregiving intensity.

### Caregiving Intensity

Caregiving intensity includes the level of dependency on the older adult and amount of care provided by the CG. CGs of racial/ethnic minority groups—African American and Latino—in particular, tend to have longer caregiving durations and CRs with more need for assistance, resulting in caregiving that is more time-intensive. For example, close to 30% of both African American and Latino CGs provide care to an older adult for 40 hr or more per week, in comparison to only 18% of non-Latino White CGs (National Alliance for Caregiving & AARP Public Policy Institute, 2015). Recent evidence from nationally representative data shows that African American and Mexican American CGs spend more time assisting with caregiving tasks than non-Latino White CGs, mostly due to high rates of co-residence with CR/CRs (Rote & Moon, 2018). Caregiving intensity is related to compromised health (Lyons, Cauley, & Fredman, 2015); however, within the CG stress process model, caregiving intensity and its effects on health may be offset by CG support.

### CG Support

Previous research has shown that CG support is related to better CG health in general (Knight & Sayegh, 2010). Formal support, or the use of paid services such as in-home or community-based care, may reduce caregiving stress, intensity, and burden and improve CG health. African American and Mexican American informal CGs tend to use fewer formal services for caregiving, and as a result rely on family and fictive kin for help with care provision (Chow, Auh, Scharlach, Lehning, & Goldstein, 2010; Crist, McEwen, Kim, & Pasvogel, 2009). However, it is important to note that even under low levels of formal care service use, there is some evidence that African American and Latino CGs—especially African American and Mexican American *dementia* CGs—report low levels of both informal support availability and satisfaction with the informal networks utilized for their caregiving services (Adams, Aranda, Kemp, & Takagi, 2002; Gelman, Tompkins, & Ihara, 2013; Janevic & Connell, 2001; Mendez-Luck & Anthony, 2015). Without the use of formal support, family-based or informal support may be especially protective against poor health for racial/ethnic minority CGs. In the CG stress process model, support is also related to attitudes toward caregiving.

### Caregiving Attitudes and Relationship Quality

Within the CG stress process model, attitudes and coping styles are also important for CG health and well-being. Despite a more demanding care situation and potentially less support, there is evidence that African American and Latino CGs express more positive attitudes toward caregiving than non-Latino Whites (Roth et al., 2015a). There is also evidence that reciprocal exchange between CG and CR is related to better mental health for African American CGs (Ejem, Bauldry, Bakitas, & Drentea, 2018); however, few studies focus on the role of CG–CR relationship quality for health by race/ethnicity. CGs who feel their CR appreciates them may experience fewer negative emotions, exhibit a lower stress response, and report better health than those with more strained relationships.

### Race/Ethnicity and Dementia Care

The nature and number of tasks associated with the CG role can be objectively difficult, especially in the case of serious cognitively impairment or dementia of the older adult. Protracted morbidity among older Mexican Americans and African Americans means that CGs face a longer period of demands (Angel, Angel, & Hill, 2014a). In addition, the severely low levels of formal care services use for African American and Latino dementia CGs and low levels of support received from family and friends (Adams et al., 2002; Gelman et al., 2013) may mean the existence of these resources is especially beneficial to health and well-being. However, there is also evidence that African American CGs are resilient in the face of dementia care and cope quite well (Haley et al., 1995). Studies on Latino CGs show that CGs face challenges in meeting the needs of their CR especially when CGs attribute dementia-related symptoms to other causes than dementia such as personality changes or normal aging processes, which can interfere with seeking support (Hinton, Franz, Yeo, & Levkoff, 2005; Rote, Angel, & Hinton, 2019). Therefore, social support and positive relationship quality may be associated with better health, especially for racial/ethnic minority CGs.

### The Current Study

The present study examines factors related to CG health across non-Latino Whites, Blacks, and Mexican Americans, the fastest growing minority ethnic group in the United States. We focus on two outcomes, distress and self-rated health, due to potential differences in manifestations of stress by caregiving factors. We expect to find that caregiving intensity will be related to poor health for all CGs, and informal and formal support will be especially beneficial for racial/ethnic minority CG health. We also expect to find that better relationship quality will be related to better health for all CGs and especially for Latino and African American CGs. Finally, we expect that because dementia care is more time-intense, having social support and a positive relationship quality will be especially important for Mexican American and African American dementia CGs.

## Research Design and Methods

We use data from Round 5 (2015) of the NSOC, which is a supplement to the National Health and Aging Trends Study (NHATS), a nationally representative study of Medicare beneficiaries 65 years and older. The NSOC consists of a sample of 2,204 informal CGs identified by the 8,334 NHATS participants in 2015. Data collection procedures and variable definitions are described in the NSOC User Guide (Kasper, Freedman, & Spillman, 2017). The NSOC obtains information on CG health and background, as well as assistance provided to the older CR. To retain CGs to community-dwelling as opposed to institutionalized older adults, we determine residential status at Round 5, or—if missing—Round 4 (2014) of the NHATS. Overall, 374 CGs to older CRs living in residential care settings were excluded.

NHATS older adult respondents were asked to provide contact information for up to five CGs who were then interviewed for the NSOC. For CRs with multiple CGs, we identified the “primary” CG as the individual performing the most caregiving duties (defined as the most caregiving hours per day), and retained them in the sample (*n* = 1,434). We also retained participants who had provided personal or instrumental care in the past month (*n* = 1,116). From this sample, missingness was most common on dementia status (*n* = 119), years providing care (*n* = 126), and CG co-residence defined as whether the CG lives with the CR (*n* = 127). Due to the limited sample size, we also dropped Latino CGs (*n* = 34) and CGs who did not identify as non-Latino White or African American (*n* = 22). The final analytic sample for CGs in the NSOC with complete information on all study variables is *n* = 667.

For Mexican American CGs, we use data from the H-EPESE CG supplement, 2016. The H-EPESE CG supplement was designed to include the same questions and response options from the NSOC to allow for comparisons between the two data sets. In the H-EPESE, older Mexican American adult participants are asked the name and contact information of the person they rely on the most for help and support. From this information, 460 CGs were interviewed about their caregiving situation and the health of the older CR. Similar to the NSOC analytic sample, we dropped participants who had not provided personal care or instrumental care in the past month (*n* = 144). Missingness was most common on years providing care (*n* = 10) and CG–CR relationship quality (*n* = 14). The final analytic sample of Mexican American CGs with complete information on all study variables is *n* = 287. It is important to note that approximately 63% of CGs in the study took the interview in Spanish.

### Dependent Variables

#### CG health

*Poor self-rated health* compares CGs who rate their health as excellent to good (=0), and fair to poor (=1). *Distress* is based on whether the respondent felt down or depressed in the past month rarely or not at all (=0) and some of the time/several days or more (=1).

### Independent Variables

#### Caregiving intensity

*Dementia* is based on whether the CR has Alzheimer’s disease, dementia, or memory problems. *Lives Together* is based on whether the CG and CR reside in the same household. *Personal Care* is based on how often the CG provided personal care to the CR in the past month (daily or not). *Years Caregiving* measures how long the CG has provided care (within the past 2 years, 3–5 years, or 6 or more years).

#### CG support

*Informal support* is based on whether the CG has family or friends that help in the care of the older adult (yes or no)*. Formal support* includes whether in the past year the CG used services that took care of the CR, so the CG could take time away from helping (yes or no).

Caregiver–Care Recipient Relationship Quality is based on the degree to which the CG feels as though the *care recipient appreciates what they do for them* (a lot = 1, other = 0). CGs are also asked how often the *care recipient gets on their nerves* (some or a lot = 1, other = 0).

#### CG background

*Caregiver–Care Recipient Relationship Type* is based on whether the CR is a child (reference category), spouse, other family, or nonfamily member. Background characteristics include CG self-reported *race/ethnicity* (African American, non-Latino White, or Mexican American), *age* (in years), *gender*, *education* (less than high school, high school, or more than high school), and *Medicaid receipt* (yes or no).

### Analytic Strategy

First, we present descriptive statistics of CG health and CG stress and coping factors stratified by race/ethnicity (Table 1). Then, we present odds ratios with 95% confidence intervals (CIs) from logistic regression analyses of CG self-reported health and distress by caregiving intensity, CG support, CG–CR relationship quality, and CG background factors in models by race/ethnicity (Table 2). In these analyses, results from the NSOC (Weights are not available for the Hispanic EPESE CG supplement.) are weighted using the *svy* command in STATA15 (for more information on weights, see: Freedman, DeMatteis, & Kasper, 2019). In the final step, we focus on a subsample of dementia CGs to identify factors related to CG health for each racial/ethnic group (Table 3).

**Table 1.** Caregiving Experience by Race/Ethnicity

	White^a^	Mexican American^b^	African American^a^
*N*	449	287	218
Health			
Poor self-rated health	23	46	19
Distress	32	37	26
CG intensity			
Dementia	25	35	23
Lives together	64	60	53
Daily personal care	16	38	21
Years care			
0 to 2 years	18	34	19
3 to 5 years	39	28	40
6 or more years	43	38	41
CG support			
Informal support	57	45	70
Formal support	18	14	17
Relationship quality			
CR appreciates CG	84	84	89
CR gets on CGs nerves	46	23	22
CG–CR relationship			
Adult child	44	62	54
Spouse	44	8	18
Other family	7	16	21
Nonfamily	6	14	8
CG background			
Age (*M*, *SD*)	66.33 (13.07)	58.76 (12.53)	58.03 (14.07)
Female	59	82	67
Education			
Less than	9	40	19
HS	29	32	22
More than HS	62	28	59
Medicaid	11	22	17

*Note.* CG = caregiver; CR = care recipient; HS = high school.

^a^National Study of Caregiving (NSOC), weighted data.

^b^Hispanic Established Populations for the Epidemiologic Studies of the Elderly (H-EPESE).

**Table 2.** Logistic Regression of CG Health by Race/Ethnicity (NSOC, 2015 & H-EPESE, 2016)

	Poor self-rated health			Distress		
	White^a^	Mexican American^b^	African American^a^	White^a^	Mexican American^b^	African American^a^
Dementia	0.40* (0.18–0.86)	0.67 (0.38–1.20)	0.72 (0.25–2.06)	0.83 (0.47–1.47)	1.28 (0.70–2.34)	2.02 (0.83–4.94)
Lives together	1.26 (0.58–2.76)	0.83 (0.46–1.49)	0.63 (0.21–1.89)	0.67 (0.29–1.55)	1.16 (0.62–2.16)	0.95 (0.42–2.15)
Daily personal care	1.19 (0.56–2.57)	1.07 (0.60–1.91)	1.01 (0.39–2.64)	1.66 (0.09–3.06)	1.14 (0.57–2.26)	0.63 (0.23–1.72)
Years care (vs 0 to 2)						
3 to 5 years	1.72 (0.68–4.33)	1.15 (0.60–2.21)	0.76 (0.30–1.90)	1.05 (0.58–1.91)	1.14 (0.57–2.26)	0.81 (0.16–4.01)
6 or more years	2.28* (1.03–5.07)	1.51 (0.81–2.81)	1.26 (0.46–3.44)	1.01 (0.54–1.91)	1.20 (0.63–2.28)	1.44 (0.37–5.52)
Informal support	1.02 (0.45–2.29)	1.24 (0.72–2.13)	1.19 (0.54–2.65)	0.69 (0.41–1.18)	0.95 (0.53–1.69)	0.78 (0.32–1.91)
Formal support	0.59 (0.26–1.35)	1.47 (0.69–3.16)	1.88 (0.82–4.27)	0.78 (0.43–1.39)	2.83* (1.29–6.20)	0.37 (0.12–1.12)
CR appreciates CG	0.28** (0.12–0.67)	0.94 (0.44–1.97)	0.17** (0.06–0.47)	0.33** (0.17–0.64)	0.82 (0.37–1.78)	0.37* (0.16–0.85)
CR gets on CGs nerves	1.61 (0.91–2.84)	1.23 (0.64–2.37)	1.29 (0.60–2.78)	2.23* (1.37–3.64)	1.68 (0.85–3.33)	1.15 (0.42–3.14)
CG–CR relationship (vs child)						
Spouse	0.84 (0.37–1.92)	2.23 (0.65–7.69)	0.35 (0.08–1.63)	1.52 (0.55–4.19)	0.39 (0.11–1.43)	1.48 (0.50–4.38)
Other family	2.21 (0.71–6.91)	1.04 (0.49–2.17)	0.26* (0.07–0.99)	0.54 (0.19–1.50)	0.47 (0.20–1.07)	0.95 (0.37–2.43)
Nonfamily	0.14 (0.012–1.54)	1.22 (0.53–2.81)	0.65 (0.12–3.46)	0.33 (0.077–1.40)	1.52 (0.64–3.61)	2.01 (0.42–9.75)
CG age	1.01 (0.98–1.04)	1.02 (0.99–1.04)	1.03 (1.00–1.06)	1.00 (0.98–1.03)	1.01 (0.98–1.04)	1.01 (0.99–1.03)
Female	0.83 (0.46–1.50)	0.40* (0.20–0.81)	0.74 (0.32–1.72)	2.44** (1.44–4.12)	1.34 (0.63–2.85)	1.57 (0.52–4.72)
Education (vs less than HS)						
HS	0.81 (0.32–2.08)	1.60 (0.85–3.01)	0.27* (0.09–0.87)	0.80 (0.32–2.02)	1.27 (0.66–2.46)	1.50 (0.50–4.56)
More than HS	0.83 (0.31–2.21)	1.11 (0.57–2.18)	0.19** (0.07–0.49)	0.62 (0.28–1.34)	0.88 (0.43–1.79)	0.97 (0.37–2.53)
Medicaid	5.59*** (2.33–13.4)	2.99** (1.57- 5.69)	5.16** (1.89–14.1)	0.78 (0.29–2.07)	4.03*** (2.07–7.84)	1.95 (0.81–4.68)

*Note.* CG = caregiver; CR = care recipient; HS = high school. 95% CI in parentheses. *N*_White_ = 449, *N*_Mexican American_ = 287, *N*_African American_ = 218.

^a^Using weighted data, National Study of Caregiving (NSOC).

^b^Hispanic Established Populations for the Epidemiologic Studies of the Elderly (H-EPESE).

*p < .05. **p < .01. ***p < .001.

**Table 3.** Logistic Regression of Dementia CG Self-Rated Health and Distress by Race/Ethnicity (NSOC, 2015 & H-EPESE, 2016)

	Poor self-rated health			Distress		
	White^a^	Mexican American^b^	African American^a^	White^a^	Mexican American^b^	African American^a^
Lives together	2.03 (0.44–9.50)	1.72 (0.62–4.72)	0.17 (0.015–2.01)	1.03 (0.39–2.75)	1.70 (0.61–4.75)	0.42 (0.10–1.72)
Daily personal care	2.04 (0.52–7.94)	1.11 (0.39–3.19)	2.65 (0.16–44.2)	1.34 (0.47–3.84)	2.86 (0.93–8.82)	0.92 (0.21–4.09)
Years care (vs 0 to 2)						
3 to 5 years	2.86 (0.44–18.5)	2.46 (0.67–9.02)	0.01* (0.0003–0.45)	2.41 (0.61–9.46)	0.85 (0.24–3.02)	0.86 (0.13–5.65)
6 or more years	4.25 (0.65–27.7)	2.33 (0.61–8.90)	0.091 (0.01–1.28)	2.03 (0.57–7.24)	0.53 (0.15–1.92)	0.80 (0.16–4.07)
Informal support	2.05 (0.54–7.78)	2.21 (0.81–6.02)	0.03* (0.002–0.56)	2.13 (0.87–5.23)	1.90 (0.72–5.05)	0.36 (0.078–1.69)
Formal support	0.53 (0.14–2.01)	1.21 (0.37–3.92)	2.19 (0.21–22.7)	1.36 (0.44–4.15)	1.14 (0.36–3.56)	0.31 (0.060–1.65)
CR appreciates CG	0.13*** (0.042–0.39)	1.04 (0.98–1.07)	1.63 (0.11–24.4)	0.19** (0.064–0.56)	0.82 (0.21–3.25)	0.15 (0.018–1.21)
CR gets on CGs nerves	0.70 (0.20–2.41)	2.35 (0.76–7.32)	1.69 (0.18–15.8)	1.07 (0.39–2.94)	1.72 (0.55–5.35)	0.41 (0.056–3.06)

*Note.* CG = caregiver; CR = care recipient; HS = high school. 95% CI in parentheses. *N*_White_ = 133, *N*_Mexican American_ = 100, *N*_African American_ = 70.

^a^Using weighted data, National Study of Caregiving (NSOC).

^b^Hispanic Established Populations for the Epidemiologic Studies of the Elderly (H-EPESE).

*p < .05. **p < .01. ***p < .001.

## Results

Table 1 shows CG self-rated health and distress by race/ethnicity. Mexican-origin CGs in the H-EPESE tend to report worse health than both White and African American CGs. For caregiving intensity, a larger portion of Mexican American CGs are dementia CGs, and recently transitioned to the CG role, in comparison to White or African American CGs. About 63% of White CGs live with their CR, in comparison to 60% of Mexican American CGs and 53% of African American CGs, reflecting the large portion of White spousal CGs in the data. Even with lower rates of co-residence, Mexican American and African American CGs report more daily care provision than White CGs.

For support, 70% of African American CGs report receiving help with caregiving from family and friends, in comparison to 57% and 45% of White and Mexican American CGs, respectively. While less than 20% of the sample utilizes formal, paid help with caregiving, Mexican American CGs have the lowest level of formal care utilization overall at 14%. Over 80% of all CGs report their CR appreciates their help a lot, but close to half of White CGs report that their CR gets on their nerves some or a lot of the time in comparison to less than a quarter of Mexican American and African American CGs. Background factors are in the expected directions with Mexican American and African American CGs being younger, reporting lower formal educational attainment, more Medicaid receipt, and being female in comparison to White CGs.

### Self-Rated Health

The first three columns in Table 2 present logistic regression analyses for poor self-rated health. First, we find that caregiving intensity is only significant for self-rated health in the model for White CGs. In particular, White CGs with longer caregiving durations (6 or more years) report a 2.28 times greater risk of poor self-rated health than CGs who started caregiving in the past 2 years (95% CI: 1.03–5.07). Surprisingly, we also find that White dementia CGs report 60% lower odds of poor self-rated health than nondementia CGs (95% CI: 0.18–0.86).

Second, we find that informal and formal support are not significantly related to self-rated health for White, Mexican American, or African American CGs. However, we do find that positive CG–CR relationship quality directly relates to significantly better self-rated health in models for both White and African American CGs. For example, White and African American CGs who feel their CR appreciates them (vs not) have 72% and 83% lower odds of poor self-rated health, respectively. We also find that CG–CR relationship type is significantly related to self-rated health but only for African American CGs. For African American CGs, CGs to other family have 74% lower odds of poor self-rated health than adult child CGs (95% CI: 0.07–0.99).

Finally, for CG background factors, Medicaid is a risk factor for poor self-rated health, regardless of race/ethnicity. Education is significantly related to self-rated health, but only for African American CGs. African American CGs who are high school graduates or more report lower odds of poor self-rated health than African American CGs who obtained less than a high school education. For Mexican American CGs, female CGs have 60% lower odds of poor self-rated health in comparison to their male counterparts (95% CI: 0.20–0.81).

### Distress

In the next step of the analyses (Table 2), we examine the same factors for CG distress. Results reveal that caregiving intensity is not related to distress, and similar to the models for self-rated health, informal support is not related to distress. Surprisingly, formal support is significantly related to distress for Mexican American CGs, but in the opposite direction as expected. That is, Mexican American CGs with who utilized paid, formal services to help with care have 2.83 times greater odds of having distress in the past month than CGs without support (95% CI: 1.29–6.20). Supplemental analyses (not presented) reveal that 80% of Mexican American CGs who utilized services are dementia CGs in comparison to 42% who did not use formal services.

For CG–CR relationship quality, we observe similar findings as those for self-rated health. White and African American CGs who report their CR appreciates their help a lot (vs not) have a significantly lower risk for distress. Additionally, we find that negative relationship quality is also related to distress, but only for White CGs. For example, White CGs who report that their CR gets on their nerves a lot (vs not) are 2.23 times more likely to report distress (95% CI: 1.37–3.64). Furthermore, different than the models of self-rated health, CG–CR relationship type is not significantly related to distress.

Finally, Medicaid is a risk factor for distress, but only for Mexican American CGs. Mexican American CGs who receive Medicaid are at a 4.03 times greater likelihood of reporting distress than Mexican American CGs not on Medicaid (95% CI: 2.07–7.84). Finally, different from the findings for self-rated health, we find that among White CGs, women are at a 2.44 times greater likelihood of experiencing distress than their male counterparts (95% CI: 1.44–4.12). It is important to note that ancillary analyses (not presented) reveal that more White men are spousal nondementia CGs than women and that more women are adult child dementia CGs than men.

### Dementia CGs

Finally, given the unique stressors associated with dementia care, we also limited the sample to dementia CGs (Table 3). For African American dementia CGs, caregiving intensity is related to health though not in the expected direction. African American dementia CGs who have been in the CG role for 3 to 5 years report significantly better self-rated health than those with more recent transitions (within the past 2 years) to the dementia CG role. In addition, African American dementia CGs who have received support from friends and family in caregiving have 97% lower odds of poor self-rated health compared to those who do not receive help (95% CI: 2.07–7.84). For White dementia CGs, the only significant factor observed is relationship quality. White dementia CGs who report their CR appreciates their help a lot (vs not) have 87% and 81% lower odds of poor self-rated health and past month distress, respectively. For Mexican American dementia CGs, none of the proposed factors are significantly related to CG health.

## Discussion and Implications

The CG population is expected to become more diverse in the coming years (National Academies of Sciences, Engineering, and Medicine, 2016). Overall, our study utilizes population-based data to investigate a constellation of factors that precede health. Our findings support previous research in that racial/ethnic minority CGs report more time-intensive caregiving situations (Rote & Moon, 2018), use fewer formal care services (Chow et al., 2010; Crist et al., 2009), and report more positive attitudes toward caregiving (Roth et al., 2015a) in terms of better CG–CR relationship quality than non-Latino White CGs. Notably, our study also demonstrates that a larger portion of African American CGs’ report help from family and friends than Mexican American or White CGs. These trends, however, do not always translate into similar associations with CG health.

The finding that White CGs who have been in their caregiving role the longest report worse self-rated health than those who transitioned to the role within the past 2 years supports the claim that long-term stress exposure and loss of resources undermines health (Pearlin, Schieman, Fazio, & Meersman, 2005). Therefore, intervention strategies should focus on the challenges of long-term caregiving such as anticipatory grief, end of life issues, and stressors outside of the caregiving domain (e.g., financial strain and other family obligations). Individual and family counseling and support groups earlier in the caregiving career have been shown to improve well-being later in the career (Haley et al., 2008) and may also improve self-rated health. These types of interventions should also focus on fostering feelings of reciprocity and appreciation, especially for White dementia CGs and African American CGs. Previous research supports the assertion that culturally based positive appraisals protect against distress for African American CGs (Heo & Koeske, 2013) and that strength-based approaches to intervening with African American CGs that foster gratitude, appreciation, and emotional support are particularly beneficial (Dilworth-Anderson, Boswell, & Cohen, 2007).

We also find that African American dementia CGs who have been in the caregiving role from 3 to 5 years report slightly better self-rated health than those who recently transitioned within the past year. Supplemental analyses indicate that most African American dementia CGs with recent caregiving transitions tend to be adult children; therefore, they may experience initial stress following dementia diagnosis of a parent or taking on the role of CG. This finding also may reflect resiliency in that, over time, African American dementia CGs may become more adept at meeting dementia care needs and mobilizing support (Haley et al., 1996). Our study, for example, shows help from family and friends is related to better health for African American dementia CGs; therefore, increasing informal support is another important area for intervention.

Surprisingly, we find that Mexican American CGs who utilized formal support services report worse health. First, formal care is culturally less accepted for Mexican American CGs, and older Mexican Americans tend to report a preference for family-based care (Angel et al., 2014b). A greater stated preference for family support may create tensions within families who turn to formal care and negatively affect CG health. However, additional analyses indicate that most Mexican American CGs who used formal services are recent dementia CGs with time-intensive caregiving and low levels of support from family or friends. Use of formal services may also be related to poor health if the care is not culturally or linguistically appropriate. Taken together, interventions mobilizing support early in the dementia caregiving career are important for Mexican American CGs. Furthermore, we show that utilization was not significantly associated with poor health for Mexican American dementia CGs, which may be due to the type of formal support utilized. Home services may both complement cultural preferences for in-home care and supplement low levels of support reported by Mexican American CGs (Crist et al., 2009); therefore, improving CG health.

There are certain limitations of the current study. First, our data are cross-sectional, and these findings may also reflect the “healthy CG effect,” which states that healthier people are more likely to become CGs and, importantly, continue caregiving over time (Fredman et al., 2010). Selection processes may explain the better health reported by White dementia (vs nondementia) CGs and African American CGs with longer CG durations (vs shorter durations). Second, we do not include specific markers of caregiving intensity that may be more consequential for CG health such as a specific medical or self-care tasks and number of CRs. Previous research suggests that the number of CRs is related to poor health for African American CGs, underlining the importance of examining multiple caregiving roles among racial/ethnic minority CGs (Kim, Chang, Rose, & Kim, 2012). We suggest these areas for future research on Mexican American CGs.

Greater accessibility, availability, and affordability of culturally and linguistically appropriate CG interventions are needed, especially for African American and Mexican American CGs. Interventions and policies should take into consideration diversity in the caregiving experience, and facilitate increased access to both informal and formal care services. Our study adds to the growing body of literature that underscores distinctive differences between African American and Mexican American CGs, indicating that both culturally based support and coping mechanisms are varied among racial/ethnic CGs. Appropriate interventions should incorporate needs assessment of both the severity of impairment of the older adult and resources available to handle the additional responsibilities by geriatric social workers, and gerontologists involved in training primary care physicians (National Academies of Science, Engineering, and Medicine, 2016).

## Funding

National Institute on Aging (Grant/Award Number: R01 AG010939, R03 AG059107-01, U01AG032947).

## Conflict of Interest

None reported.
